# The importance of appropriate selection of clinical endpoints in outpatient COVID-19 clinical trials

**DOI:** 10.1038/s43856-023-00281-1

**Published:** 2023-04-17

**Authors:** Kristian Thorlund, Davey Smith, Christopher Linsell, Nicholas White, Christopher Butler, David Boulware, Judith Currier, Ofir Harari, Edouard Lhomme, Nathalie Strub-Wourgaft, Stacey Adam, Edward Mills

**Affiliations:** 1 McMaster University, Hamilton, Canada; 2 School of Health Sciences, University of California San Diego, San Diego, CA USA; 3 Vanderbilt Institute for Clinical and Translational Research (VICTR) Methods Program, Vanderbilt University, Nashville, TN USA; 4 Nuffield Department of Medicine, Centre for Tropical Medicine and Global Health, University of Oxford, Oxford, UK; 5 Nuffield Department of Primary Care Health Sciences, University of Oxford, Oxford, UK; 6 University of Minnesota Medical School, Minneapolis, MN USA; 7 UCLA Division of Infectious Diseases, Department of Medicine, University of California Los Angeles, Los Angeles, CA USA; 8 Cytel, Vancouver, Canada; 9 Hospital Center University De Bordeaux, Bordeaux, France; 10 Drugs for Neglected Diseases Initiative, Geneva, Switzerland; 11 Foundation for the National Institutes of Health, North Bethesda, MD USA

**Keywords:** Viral infection, Health care, Clinical trial design

## Abstract

Clinical trial endpoints must be carefully and intentionally selected so that the results of the trial can be used to inform policy- and decision-making. The relative importance of potential endpoints often depends on the stakeholder, with patients having different preferences to policymakers and regulators. The set up of clinical trials for COVID-19 was problematic, as endpoints that could be reasonably measured did not always match the efficacy endpoints usually required by guideline panels. Thus, different endpoints were used, which made the timely comparison and evaluation of interventions difficult. Here we discuss the evolution of the COVID-19 landscape and the effect this is having on the selection of consistent and measurable clinical trial endpoints. Using appropriate endpoints is crucial for researchers to offer the most reliable, valid, and interpretable results possible.

## Abstract (web summary)

Thorlund et al. discuss considerations when selecting appropriate endpoints for outpatient COVID-19 clinical trials. Improvements in patient outcomes during the COVID-19 pandemic have necessitated changes to the outcomes being assessed.

## Introduction

Clinical trial endpoints must be carefully selected to ensure that clinical trial evidence can inform decision-making, both in policy and in practice. The relative importance of potential endpoints often varies by stakeholder, with preferences differing between patients (e.g., preventing severe disease), clinicians (e.g., treatment management), policymakers (e.g., reducing costs), and regulators (e.g., patient safety). While patients may be concerned with outcomes such as recovery time, prevention of severe disease, and prevention of long-term effects, the most important issue for other stakeholders may be entirely different. Regulators, for example, may require outcomes that are comparable across multiple studies. Appropriate endpoints can also change; for example, clinical endpoints such as prevention of hospitalization and death from COVID-19 have become increasingly difficult to detect due to widespread immunization and immunity acquired from previous infection. There is now a difference between what can be reasonably measured in outpatient COVID-19 clinical trials and what guideline panels require as efficacy endpoints.

Overcoming these differing perspectives and establishing appropriate clinical trial endpoints is a critical component of study design, as how and when measurements are made define the quality of the data, and thus, how the trial results will be received, and the effect they might have in policy and medical practice. Navigating this process often involves carefully balancing stakeholder perspectives with practical considerations such as participant burden, costs, location, and logistics. Here we discuss how to determine appropriate clinical trial endpoints for COVID-19 therapeutic development.

## Outpatient trials to evaluate COVID-19 therapies

Previous trials evaluating inhaled budesonide, oral fluvoxamine, and the oral antiviral agents molnupiravir (Lagevrio™), as well as nirmatrelvir combined with ritonavir (Paxlovid™) illustrate the challenges associated with identifying appropriate endpoints, as each trial measured different outcomes that had little consistency in terms of measurement^1–4^. These outpatient clinical trials used outcomes such as change in naso- or oropharyngeal viral load densities, change in symptom severity, time to clinical improvement, emergency setting visits, hospitalization, and mortality, sometimes combining these outcomes into composite outcomes. These outcomes vary substantially in their complexity and degree of subjectivity^5^. For example, if correctly performed, a body temperature or SpO2 reading should be highly reproducible, while the measurement of how a patient feels and functions is more challenging. Similarly, an SpO2 measured in the home by a participant may not be as reproducible as one obtained in a healthcare setting. These differences prevent timely comparative evaluation of agents for patients with COVID-19 in the outpatient population.

Compared to trials among severe and critically ill patients, which are typically conducted in inpatient settings, trials of potential COVID-19 treatments in non-severe cases are typically conducted in outpatient settings and present different challenges. Of the 580 published randomized controlled trials that have evaluated interventions for COVID-19, only 61 (10%) have focused on the outpatient treatment setting, despite this being the most frequent experience for patients^6^. The relative lack of outpatient trials for treating COVID-19 may stem in part from the logistical difficulty of conducting an outpatient versus inpatient trial (e.g., difficulty with patient recruitment and follow-up) and the need for different clinical endpoints^7,8^. For example, the primary aims of treatment in patients with severe COVID-19 are to prevent the need for more invasive interventions (such as mechanical ventilation) and death, while treatment of outpatient COVID-19 primarily aims to decrease duration and severity of illness, as well as preventing disease progression, which comes with the need for additional medical care, such as hospitalization. Even for drugs that demonstrate impressive reductions in the relative risk of more traditional regulatory-approved endpoints (e.g., hospitalization, mortality), the absolute benefits of these reductions are being increasingly diminished as the overall risk of severe outcomes declines due to the emergence of less severe SARS-CoV-2 variants, the acquisition of infection- and vaccine-induced immunity, and the arrival of monoclonal antibodies that can neutralize SARS-CoV-2 and antivirals that improve the standard of care^9^. Furthermore, there is substantial heterogeneity even within the COVID-19 outpatient population, both in terms of risk factors that may affect their susceptibility to severe outcomes (e.g., age, vaccination status), and in terms of the treatments they are likely to receive. All of these variables underscore the need for differing treatment strategies and clinical endpoints that are applicable to the unique needs of the patient.

## Rethinking COVID-19 trial endpoints

**Fig. 1:**
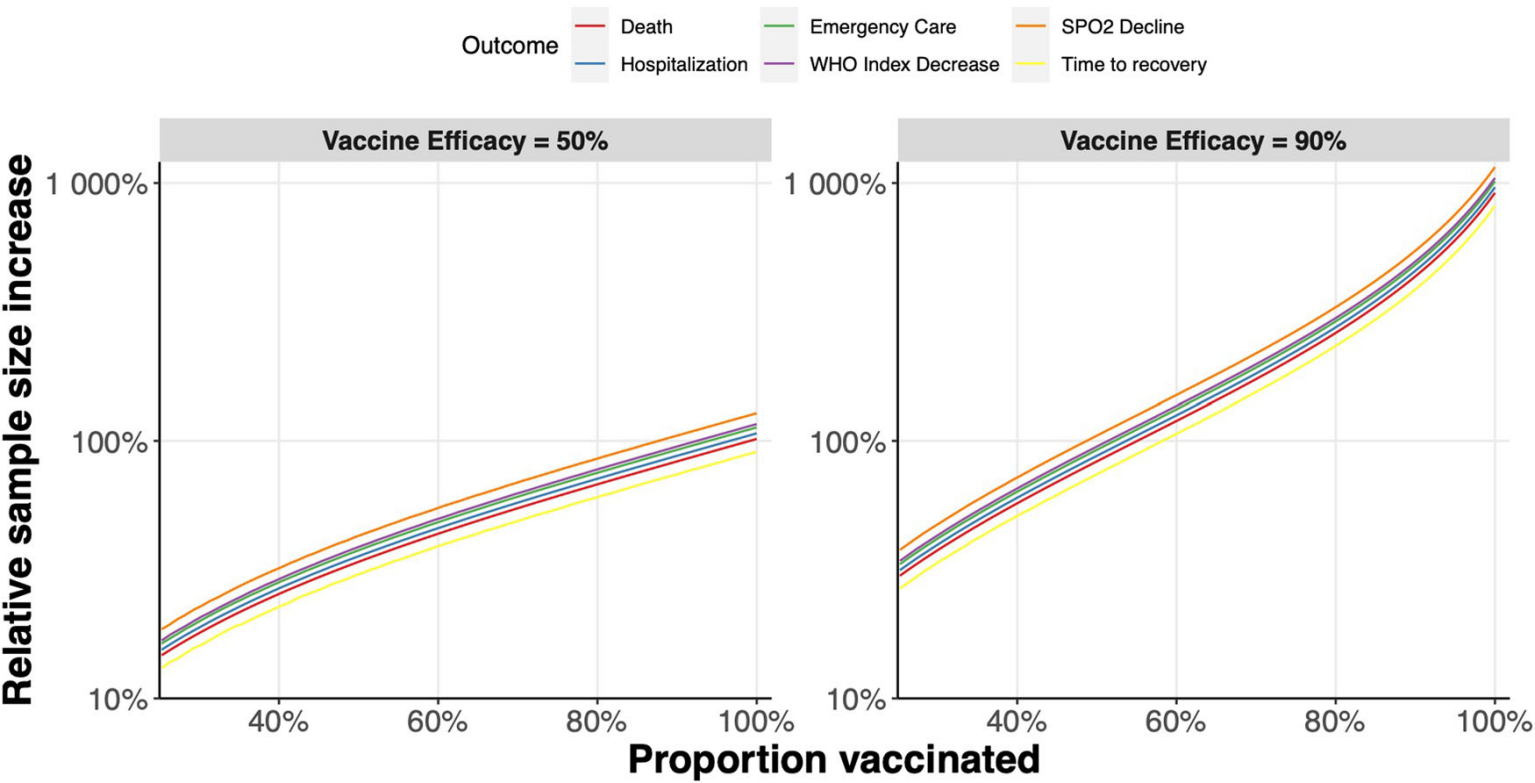
Sample size necessary for outpatient clinical trials with varied endpoint event rates and vaccine coverage. We assumed background (control) event rates (CER) of 2%, 7·5%, 13%, 16% and 25% for death, hospitalization, emergency care use, a 2-point improvement on the WHO symptom scale and SPO2 decline, respectively. 14-day recovery was assumed to occur 92·5% of the time in the control arm (all but hospitalized). We then calculated the sample size required for 90% power to detect a 30% relative risk reduction (or equivalently, 0·7 hazard ratio in the case of time to recovery) effect at the 2·5% significance level, assuming zero vaccine coverage^19,20^. The power was then held fixed at 90% and modified sample size requirements were calculated, as the background rates changed according to CER(adjusted) = CER*(1-VE*p), where VE is the vaccine efficacy and p is the vaccine coverage in the general population, under the simplifying assumption that VE remains the same for all different outcomes. The increase in sample size relative to zero vaccine coverage is shown in the figure for all outcomes.

The rapidly changing dynamics of the COVID-19 pandemic create several challenges for the design of clinical trials. Good clinical trials are designed to minimize bias and generate high-quality evidence for treatment effects across outcomes that are important to patients. Figure 1 illustrates how, as vaccination rates go up in a population and rates of severe disease decline, the power to detect statistically and clinically significant treatment effects in clinical trials decreases. As a result, clinical trials in countries with high vaccine coverage are taking longer to recruit participants. Also, event rates that are lower than initially expected result in longer trials that contribute new knowledge more slowly to the COVID-19 evidence base. For instance, the decreasing frequency of hospitalization may now compromise the feasibility of using hospitalization as an outcome. Indeed, the decreasing incidence of hospitalization may necessitate a change to more frequent outcomes, possibly even those that occur in every patient, such as duration of illness, to allow trials to be adequately powered and completed quickly enough to inform current policies.

An alternative to changing the focus to more frequently occurring outcomes could be to restrict enrollment to higher risk populations with low vaccine coverage or other vulnerabilities (e.g., elderly, immunocompromised patients, or those with comorbidities) who are expected to have a higher event rate. This, however, is insufficient, and raises potential questions regarding generalizability, especially for an agent that may be expected to be used in a population that was not included in the clinical trial. For example, older people with multiple co-morbidities may not be eligible for a clinical trial because they have received various doses of COVID-19 vaccines but would still be provided therapy in clinical practice. Furthermore, trials in largely unvaccinated populations would be nearly impossible to conduct given the widespread uptake of vaccination worldwide, and the resulting lack of generalizability of such a trial. Trials that enroll heterogenous populations increase the clinical applicability of effective therapies, so exclusions based on vaccination status, comorbidities, variant infection, location, and availability of therapies outside the trial should be avoided where possible. This highlights the challenge of maintaining traditional endpoints and increases the desirability of uniform and accurate measurement of outcomes that, while harder to assess accurately, remain relevant to patients and guideline developers and regulators.

## What outcomes should we be measuring?

Table 1 illustrates the strengths and weaknesses of common outcomes used in COVID-19 clinical trials for outpatient trial participants. The table illustrates that symptom severity and duration are becoming increasingly important in trials evaluating therapies for early COVID-19. However, ideally all trials would measure these outcomes using similar methods. The World Health Organization’s (WHO) recommended clinical progression scale for use in COVID-19 trials is of limited utility in this context, in that just four of the eleven categories relate to the outpatient setting, and of these, only three categories relate to symptoms (asymptomatic, symptomatic independent and symptomatic assistance needed)^10^. While the measure is intended to be assessed daily, its utility is limited for measuring symptom burden, severity, and duration.

**Table 1:** Potential primary endpoints in COVID-19 outpatient clinical trials.

Outcome	Strength(s)	Weakness(es)
Death	Objective Top patient priority Easy to capture	Very low proportion in outpatient setting Lower in high income country with well-resourced healthcare settings Decreasing in frequency with vaccination and less severe variants Not a feasible outcome in non-severe patients
Hospital admission	Important to patients in itself Important for the effect on healthcare system Indicative of follow-on effects Easy to capture	Between-setting variation in practice Decreasing in frequency with vaccination and less severe variants In vaccinated settings, has decreased to an extent that may no longer be a feasible outcome
Recovery (including time to recovery)	Important to patients Measurable in every patient, and thus requiring many fewer patients to detect treatment effects	Subjective, raising measurement challenges and markedly increasing importance of blinding to reduce bias
SpO_2_	Objective, standardized measurement Easy implementation	Inconsistent readings with cold digits. A surrogate, not itself important to patients.
Viral Load	Objective, standardized measurement	A surrogate, not itself important to patients Poor correlation to severe outcomes and patient experience
Symptom Severity	Ordinal or continuous scales Easy to capture	Subjective, raising measurement challenges and markedly increasing importance of blinding to reduce bias Importance of individual symptoms varies between patients
WHO Ordinal Scale	Well used Easy to capture	Includes outcomes that occur very rarely in the population and it has only two categories measurable in outpatients

## The case for a focus on important outcomes in patients with early non-severe COVID-19

The continuum of COVID-19 severity should be considered when developing treatments that aim to reduce disease burden. Patients who do not develop severe symptoms, such as shortness of breath at rest or with minimal activity, will be less likely to visit an emergency room than those who develop severe symptoms. Those who visit an emergency room will be far more likely to be admitted to hospital than those who do not visit an emergency room. Those who are admitted to hospital are candidates for mechanical ventilation, while those not admitted are not. Because severity of illness is the primary determinant of hospitalization, those admitted to hospital are far more likely to die than those not admitted to hospital. It therefore follows that reducing the frequency with which those with early COVID-19 progress to severe symptoms is very likely to reduce emergency room visits, hospitalizations, and the need for mechanical ventilation. If deaths occur very infrequently among those contracting the virus (e.g., 4 in 1000 or less), any mortality benefit from reducing symptom severity may be very small and undetectable in feasibly-sized trials. However, as it is very likely that reducing the frequency with which severe symptoms develop will ultimately reduce death, reducing symptoms should be an acceptable goal. Another, and perhaps even more compelling reason to consider symptom burden, is that while hospitalization, need for mechanical ventilation, and death are obvious harms to avoid, disease duration and severity are also important to patients. In other infectious disease scenarios, healthcare teams do not generally treat chest infections, sore throat, etc., with the goal of keeping people out of hospital; healthcare teams generally treat to help people recover more quickly. This is also the case with seasonal influenza, which also carries a risk for hospitalization, need for mechanical ventilation, and death. Healthcare providers often use a wide variety of possible remedies that aim to minimize symptom severity and duration of illness. The changing dynamics of the COVID-19 pandemic, such as the increases in infections and sick leave driven by the Omicron wave and the increasing emergence of long COVID^11^, illustrate how rapid recovery from symptoms may be of great importance.

## Reliable, valid, interpretable measures of symptom severity and duration of illness are needed

Composite outcomes as primary endpoints may be useful to improve the statistical power of outpatient clinical trials, so there is value in identifying which individual outcomes may be reliably combined to form endpoints. Clinical endpoints are by definition composite endpoints (e.g., death has various causes). Guidance for the use of composite endpoints is common in other areas of medicine, such as cardiology, but less common for infectious diseases^12^. Similarly, ordinal scale outcomes illustrating patient improvement or disease progression have been used in some COVID-19 trials. Indeed, the WHO ordinal scale represents a form of composite endpoint^13,14^. For outpatient trials in populations with increasing vaccination rates, where progression to hospitalization is unusual, and the desired societal effect has shifted to helping the patient feel better faster with full return to pre-illness health state, we recommend careful selection of the primary objective (prevent disease progression or help patients feel better faster), coupled with a more complete collection of outcomes that are meaningful to patients, policy makers, healthcare providers and others.

## The way forward

While some people with COVID-19 are still hospitalized, many now recover at home, especially those who have been vaccinated^15,16^. However, COVID-19 symptoms still affect patients’ lives. Treatments that lessen symptom severity and duration of illness are likely to reduce more severe outcomes. as those with less severe and shorter illness are almost certainly less likely to suffer severe outcomes, even if this may not be measurable given the small magnitude of effect. Since symptom severity and duration are themselves important for patients, clinical trials for early COVID-19 should use symptom severity and duration of illness as endpoints. Given that viral burden varies over the course of infection and that progression to severe illness may be more common in the later stages of infection^17^, chosen endpoints should also account for timing of assessment. The measurement of this endpoint should be agreed upon and used uniformly across trials. Such standardized measures should aim to have high relevance for patients, clinicians, policymakers, regulators, and society.

As the pandemic evolves, it will become increasingly important for treatments to be evaluated in heterogenous populations (e.g., across vaccination status, age, co-morbidities, locations). Recovery outcomes may be more important for these populations, and the importance of a specific outcome may depend on the group to which patients belong. For example, high-risk, unvaccinated people may want treatments that prevent hospitalization, while low-risk, vaccinated people may want less severe disease. Some treatments may be safe and low cost, and thus cost effective in treating symptoms. High-cost treatments with more side effects and interactions are likely to have a different primary purpose, to prevent disease progression and keep people out of hospital. For example, paxlovid is boosted with ritonavir, a cytochrome P450 inhibitor resulting in increased distribution of paxlovid, but also any other drugs metabolized by this cytochrome^18^. These interactions should be carefully considered when developing a treatment strategy. However, if a safe and low-cost method of reducing symptoms is denied approval because of a lack of evidence it prevents hospitalization, we risk denying many patients the benefit they want. By understanding the diverse needs of different groups of stakeholders, outpatient trials can achieve both relevance and scientific reliability across the multiple patient risk groups they will enroll.
